# Dated phylogeny suggests early Neolithic origin of Sino-Tibetan languages

**DOI:** 10.1038/s41598-020-77404-4

**Published:** 2020-11-27

**Authors:** Hanzhi Zhang, Ting Ji, Mark Pagel, Ruth Mace

**Affiliations:** 1 Department of Anthropology, University College London, London, WC1H 0BW UK; 2 Key Laboratory of Animal Ecology and Conservation Biology, Centre for Computational and Evolutionary Biology, Institute of Zoology, Chinese Academy of Sciences, Beijing, 100101 China; 3 School of Biological Sciences, University of Reading, Reading, RG6 6UR UK; 4 Santa Fe Institute, Santa Fe, NM 87501 USA

**Keywords:** Anthropology, Cultural evolution, Phylogenetics

## Abstract

An accurate reconstruction of Sino-Tibetan language evolution would greatly advance our understanding of East Asian population history. Two recent phylogenetic studies attempted to do so but several of their conclusions are different from each other. Here we reconstruct the phylogeny of the Sino-Tibetan language family, using Bayesian computational methods applied to a larger and linguistically more diverse sample. Our results confirm previous work in finding that the ancestral Sino-Tibetans first split into Sinitic and Tibeto-Burman clades, and support the existence of key internal relationships. But we find that the initial divergence of this group occurred earlier than previously suggested, at approximately 8000 years before the present, coinciding with the onset of millet-based agriculture and significant environmental changes in the Yellow River region. Our findings illustrate that key aspects of phylogenetic history can be replicated in this complex language family, and calls for a more nuanced understanding of the first Sino-Tibetan speakers in relation to the “early farming dispersal” theory of language evolution.

## Introduction

Sino-Tibetan languages make up the second-largest language family in the world^1^ comprising around 500 languages that stretch from the western Pacific to the Himalayas, Nepal and India-Pakistan in the west, and account for around 1.4 billion of the world’s speakers. A long history of frequent and often intimate contact with speakers of other language families (e.g. Austroasiatic, Tai-Kadai, Hmong-Mien, Austronesian, Altaic) and complex histories of population migration have meant that Sino-Tibetan languages exhibit complex morphologies which have posed challenges to traditional linguistic comparative studies designed to understand the origins and genealogical relationships among the Sino-Tibetan languages^2^. Using traditional methods, many linguists favour the ‘farming dispersal’ hypothesis^3^, proposing that Sino-Tibetan languages arose in agricultural societies in Northern China (e.g. Yangshao culture) around 6500 BP and expanded westwards into the Himalayas with the dispersal of millet agriculture^2,4,5^. According to this “Northern China origin” hypothesis, the Sinitic or Chinese languages form the primary branch near the root of Sino-Tibetan tree^6–8^.

An alternative to the “Northern China origin” proposal is that the ancestral Sino-Tibetan speakers were early Neolithic populations from Sichuan who migrated westward to the Lower Brahmapūtra basin before 9000 BP then eastward to the Yellow River basin around 8000 BP^9^. More recently, some linguists have suggested that the earliest speakers of Sino-Tibetan were highly diverse foragers living in the eastern Himalayas before 9000 BP who migrated westwards to the high Tibetan Plateau after 7500 BP and later eastwards to China by 5000 BP^10^. Both the “Eastern Himalayan origin” and the “Sichuan origin” hypothesis, expect that the Sino-Tibetan phylogeny will have a rake-like topology where all subgroups evolved independently from each other; Sinitic and Bodish clades are predicted to be closely related to each other and form a lower level subgroup among other languages of secondary migratory populations^10–12^.

The development of statistically-based Bayesian phylogenetic inference methods makes it possible formally to test among the various theories for the origin of the Sino-Tibetan languages. Unlike traditional comparative linguistic studies that compare morphological features, Bayesian inference methods applied to linguistic data compare cognates of core vocabulary that are relatively resistant to horizontal borrowings^13^. Languages may be especially useful for studying modern human cultural history because the pace of most genetic evolution can be too slow to resolve relatively recent events^14^. In addition, where there has been, as with the Sino-Tibetans, a long history of migrations, the genetic historical signal can actually obscure the relevant cultural-linguistic history because cultures (and their languages) can often remain relatively stable in the face of genetic immigration^15^.

In agreement with the “early farming dispersal” hypothesis^16^, two recent Bayesian phylogenetic studies of the Sino-Tibetans^17,18^, using independently derived linguistic datasets, find evidence that Sinitic languages do indeed form the primary branch near the root of the Sino-Tibetan tree and suggest that ancestral Sino-Tibetans were millet farmers from Northern China. But the two studies estimate different timings for the initial Sino-Tibetan divergence, 5871 BP^17^ versus 7184 BP^18^, and yield different phylogenetic relationships among subgroups and time-depths of subgroup formation (see Table S3-4). To address these uncertainties in Sino-Tibetan language evolution, we investigate the phylogeny of the Sino-Tibetan languages using a third lexical dataset based on a larger and linguistically more diverse sample (Methods, Tables S6-S8). This provides an unusual opportunity to examine the credibility and generalizability of the Sino-Tibetan phylogeny’s features, and comes at a time when the importance of independent replication of scientific findings is increasingly recognised^19^, especially in the human sciences^20^.

**Figure 1:**
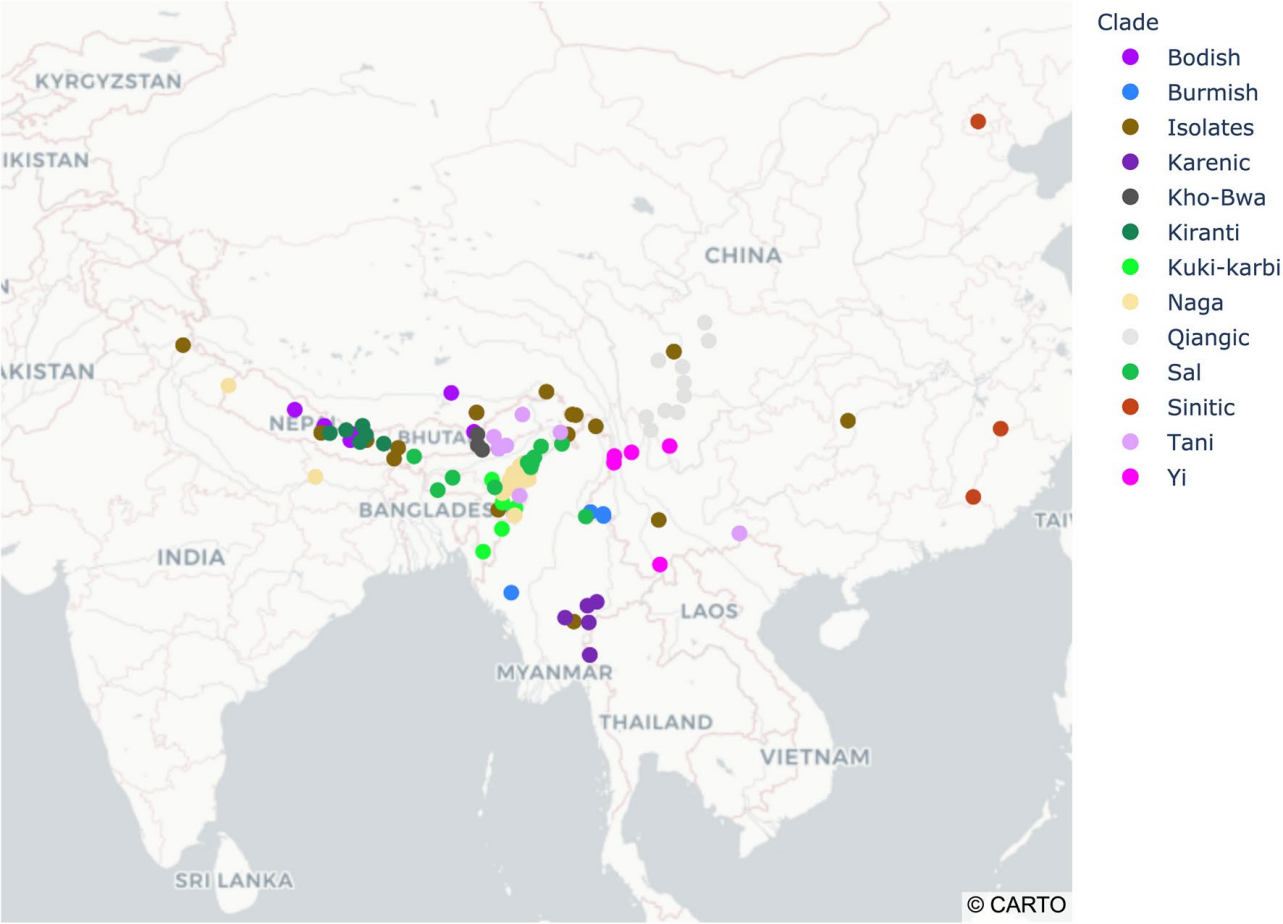
Geographical distribution of major clades of the 131 Sino-Tibetan languages sampled in this study, as annotated in the Maximum Clade Credibility tree diagram (Fig. 2).

Our dataset comprises information on shared cognates for 110 items of vocabulary for 131 Sino-Tibetan languages (Fig. 1), and makes use of calibration points taken from written historical records. We analyse these data using Bayesian phylogenetic inference methods that, in combination with calibration points, allow us to infer a time-calibrated phylogenetic tree. The statistical approach makes it possible directly to assess the strength of support for alternative phylogenies, including hypotheses about the most probable outgroup to the Sino-Tibetans, the timing of the origin of this language family and the support for relationships among its major clades.

## Results

### Dated phylogeny of Sino-Tibetan languages

Our data yielded 1726 binary cognate sets distributed over the 110 lexical items (Methods). We inferred time-calibrated phylogenetic trees of the Sino-Tibetans from these data, comparing several models to characterise cognate class evolution (Methods, Table S2). Our analyses used a relaxed-clock approach that allows the rate of lexical evolution to vary throughout the tree^21^, and we employed a fossilized birth–death tree prior^22^ as implemented in BEAST2^23^. This tree prior is appropriate for time-structured data containing taxa some of which do not survive to the present, and makes no assumptions about population sizes or their stability throughout the time period covered by the tree.

We calibrated the tree using historical records (Table S1), including extinction times for the historical languages (Old Chinese, Padam, Shaiyang). We calibrated the most recent common ancestor of Lolo-Burmese languages, of Pumi languages, and of Naxi languages, to be earlier than the date when their descendants were described in written historical records. Unlike in one previous phylogenetic study of the Sino-Tibetans^17^, our calibrations are based solely on these empirical records rather than traditional linguistic theories.

The covarion model^24^ emerged as the best supported model of cognate class evolution (Table S2), as in previous studies of this group and we use it in all analyses reported below. To identify the outgroup of Sino-Tibetan phylogeny, we first performed inferences on our data without any monophyletic constraint on the outgroup. Similar to the two previous studies of the Sino-Tibetans, the unconstrained analyses found the Sinitic clade as the best supported outgroup, occurring in our data with 80.13% posterior probability, followed by the second candidate (Sinitic + Sal + Tani + Kiranti + Kho-Bwa clades) with a much lower posterior probability of 14.32%. Inferences with the outgroup constrained to be Sinitic are better-fitting than inferences without outgroup constraints (Bayes Factor = 20.18, Table S5). Based on these findings, we performed all further phylogenetic inferences constraining the Sinitic clade to be the outgroup (for diagnostics and the reconstructed phylogeny without outgroup constraint, see Table S5 and Figure S4). We did not place any further monophyletic constraints on the tree.

**Figure 2:**
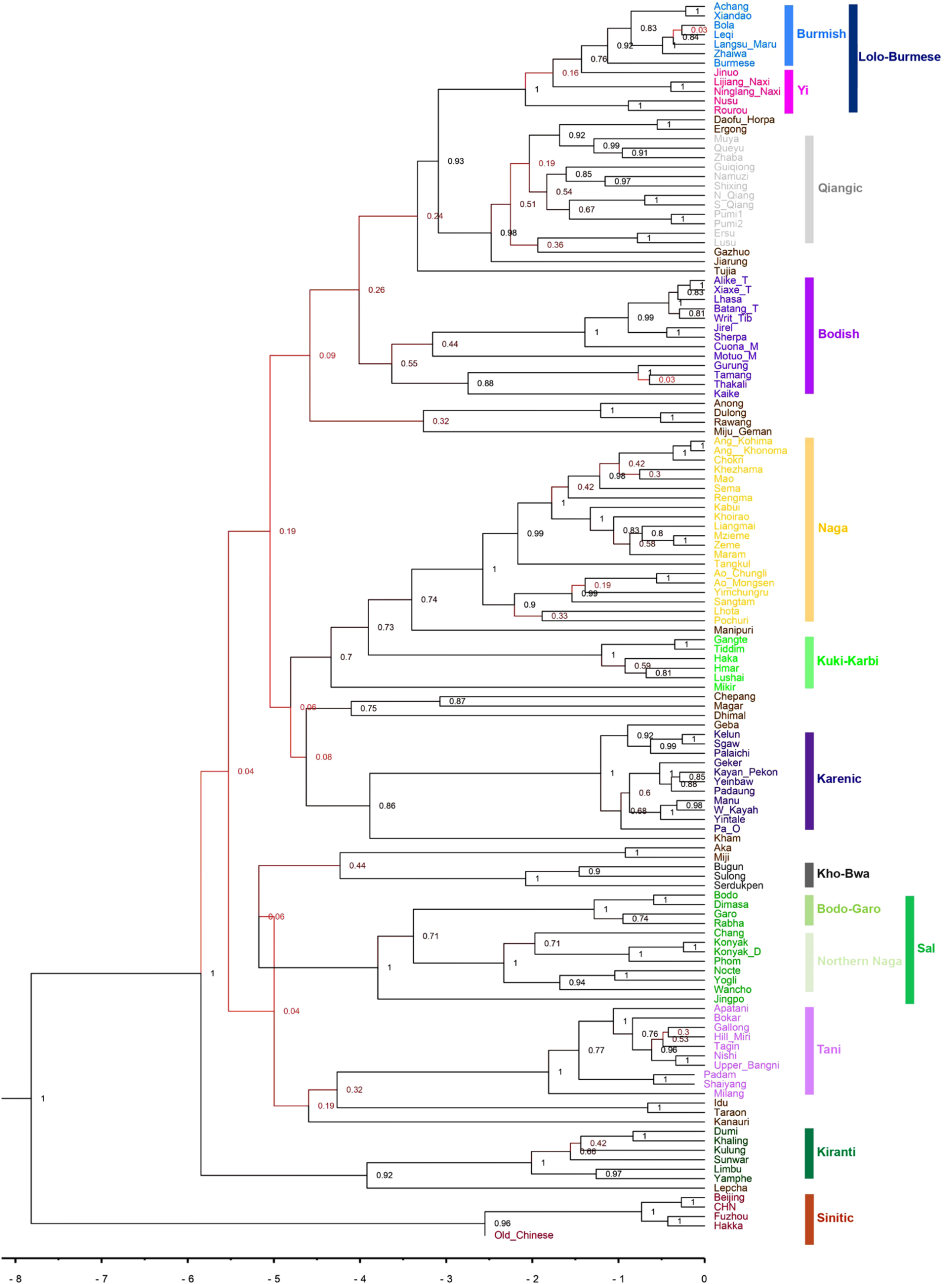
Maximum Clade Credibility tree of 131 Sino-Tibetan languages sampled in this study, inferred with relaxed clock and covarion model using the Sinitic clade fixed as the outgroup. Posterior probabilities of internal nodes are shown. The time scale is in units of thousand-of-years before the present. See Figure S4 for the reconstructed phylogeny without an outgroup constraint.

The time-calibrated phylogenetic tree of the Sino-Tibetans (Fig. 2) yields posterior support with greater than 95% probability for ten independent subgroups: Lolo-Burmese, Qiangic, Bodish, Naga, Kuki-Karbi, Karenic, Kho-Bwa, Sal, Tani, and Kiranti. Unlike previous studies, we did not find posterior support for Tibeto-Dulong, Tani-Idu, or Tibeto-Gralrongic as independent subgroups (see Table S3). We infer mean ages of these subgroups similar to two previous computational phylogenetic studies^17,18^. We estimate the mean root age at 7983 years BP, with a 95% highest posterior density interval of 4778–11,285 BP. The root represents the first divergence event of the proto-Sino-Tibetan language ancestral to all extant Sino-Tibetan languages sampled. Our inference of the mean root age and the other time depths using the Sinitic outgroup constraint are nearly identical to inferences without any outgroup constraint (Figure S2-3).

### Phylogenetic topology and linguistic taxonomy

Linguists have differing opinions on several features of the internal topology of the Sino-Tibetan language tree. Kiranti languages are thought by some to be closely related to Magar, Kham, and Chepang^17,25^. However, we find (Fig. 2) that Kiranti forms a distinct subgroup (posterior probability = 0.92), independent of other Himalayan languages, as has been previously proposed^26^, and that it is unlikely to originate from the same ancestor as Magar, Kham, and Chepang (posterior probability = 0.02, see Table S3). Tani languages share some similarities to Taraon and Idu and there is uncertainty among linguists as to whether this arises from common descent or contact and borrowing^8^. We find little support for Tani languages to form a subgroup with Taraon and Idu (posterior probability = 0.32).

We do not find support for the view^8^ that Dulong is closely related to Gyalrong (Jiarong) and Qiangic languages, and we find only weak support for the view that the Bodish and Lolo-Qiangic languages form an independent subclade (posterior probability = 0.40). By comparison we do find that Lolo-Burmese and Qiangic languages are closely related (posterior probability = 0.95), and that Bodo, Konyak, and Jingpo languages can be classified into a single subgroup ‘Sal’^27^. Nevertheless, our result showed very little support for the classification of Sal languages as a separate branch from all other Tibeto-Burman languages^28^.

The “Eastern Himalayan origin” hypothesis proposes that the prehistory of Sino-Tibetan languages is characterized by a prolonged parallel evolution of Himalayan subgroups and that Sinitic languages differentiated from Bodish and Lolo-Qiangic languages recently. Although our inference supports the scenario of Himalayan subgroups evolving in parallel, there is no evidence that Himalayan languages were ancestral to Sinitic languages^10^. The “Sichuan origin” hypothesis proposes a deep dichotomy between a Northern clade of Sinitic and Bodish languages, and a Southern clade of Lolo-Qiangic and Karenic languages^9^. Our inferences do not support this topology. Both the “Eastern Himalayan origin” and “Sichuan origin” hypotheses propose that Kuki-karbi is the most likely outgroup of Sino-Tibetan phylogeny and predict Sinitic and Bodish languages to be closely related to each other^11,12^. We found no evidence of a Kuki-karbi outgroup or a Sino-Bodish subgroup.

According to the “Northern China origin” hypothesis, the Sinitic languages form the primary branch near the root of Sino-Tibetan tree and all non-Sinitic languages descended from an ancient common ancestor (i.e. proto-Tibeto-Burman)^6–8^. Previously^17^, the initial divergence of Sino-Tibetan languages was associated with the geographic spread of millet agriculture from the Yellow River basin, based on the inferred age of Sino-Tibetan phylogenies. Here our inference replicates an early bifurcation into the Sinitic clade and the Tibeto-Burman clade and Sinitic languages forming the primary branch near the root. Nonetheless, our estimated date of initial divergence suggests that the first Sino-Tibetan speakers were more likely to be growing populations of incipient agriculturalists, rather than out-migrating groups of specialised agriculturalists.

## Discussion

We find that the Sinitic and Tibeto-Burman languages first began to diverge during the early Neolithic, at approximately 8000 years BP, earlier than previous estimates for this group^17,18^, although 95% posterior density intervals of all three studies overlap. Our greater time-depth probably owes to our sample containing a wider range of linguistic taxa than previously studied, including Naga, Kho-Bwa, Karenic, Konyak – languages that are distantly-related to Sinitic languages. Our inferred date of 8000 years BP for the initial divergence between Sinitic and Tibeto-Burman languages coincides with the onset of millet-based agriculture in the Yellow River region (circa 8100–7700 BP)^29^, and a period of significant environmental change from cold-dry (11,000–8700 BP) to warm-wet conditions (8700–5500 BP) in North-central China^30,31^ and in South China (10,400–6000 BP)^32^. Recent study found a substantial population growth in Neolithic northern China started in the late seventh millennium BCE, which was likely initiated by the onset of millet-based agriculture^29^. Recent palaeoecological studies with high-resolution data also showed that, in North-central China, the transition to warm-wet climate took place at 8100–7900 BP, followed by a rapid development of sedentism and social complexity^33,34^.

On average, our reconstructed phylogeny showed that first Bodish speakers were present circa 5000 BP and Bodish languages began to diverge around 3600 BP, consistent with the archaeological evidence that modern humans settled extensively in the northeastern Tibetan Plateau with millet cultivation around 5200 BP, and further expanded to high-altitude plateau areas 3600 BP with barley and sheep^35^. Our inference is also consistent with the genetic finding that proto-Tibeto-Burman populations experienced large population expansion from 4.2 to 7.5 thousands years ago^36^.

Archaeological records suggest there was a marked population growth during the sixth millennium BC (8000 BP) in the middle Yangzi region^37^. From 8000 to 4000 BP, archaeological records suggest a 50-fold increase in population in the Yellow River valley^38^. Nevertheless, the strongest evidence for large scale migration by Neolithic farming populations in North-central China is from 6500 to 4500 BP^39,40^. Ancient-DNA analyses also suggest that early Neolithic farmers in North China did not expand into southeast China until after around 6000 BP^41^. Although millet domestication began in North China as early as 10,000 years BP^42^, archaeological records show that subsistence strategies relied heavily on foraging and plant domestication played rather minor roles in subsistence during the early Neolithic period^43,44^.

This calls for a more cautious interpretation of the inferred root age, and a more nuanced understanding of the first Sino-Tibetan speakers than ‘out-migrating farmers’^16^. A previous study^17^ associated the initial divergence of Sino-Tibetan languages with the geographical spread of millet agriculture. However, the trigger for language divergence processes was not necessarily migration or geographical separation. The inferred root age (initial divergence date) likely represents the formation of subgroups of speakers separated by distinct ecological niches or social distances, who are no longer in frequent contact and thus start to innovate their language in different ways. Unlike farming dispersals in western Eurasia, where farmers with Middle Eastern ancestry largely replaced hunter-gatherers in Europe^45^, farming in East Asia may have spread gradually through the mixing of farmers and hunter-gatherers.

A more nuanced version of the ‘early farming dispersal’ hypothesis^46^ recognises that prehistoric language expansions did not occur when the first settled agricultural societies arose but only after a suite of food production and domestication practices coalesced into a mobile agricultural package which would follow the migrating populations into new territories. In the Sino-Tibetan region, this means adaptation to the mountainous terrain of Southwest China and the high altitude of the Tibetan Plateau and the Himalayas which is a prolonged process over millennia. In North-central China, it took two to three millennia for the development of agriculture and animal domestication to raise population size sufficiently for demic spread to occur^47^.

The evolutionary history of Sino-Tibetan populations is complex and mosaic. Both archaeological and genetic studies suggested that the initial occupation of the Tibetan plateau was followed by multiple migrations at different times and from different places^48^. Whole-genome sequence data estimate that modern Tibetan and Han Chinese populations diverged from their shared ancestral population circa 15,000 to 9000 BP^49^. There is archaeological and genetic evidence for subsequent waves of migrations of Neolithic millet farmers to the Himalayas during the mid-Holocene^35,50–53^. Both demic and cultural diffusions might have occurred during the transition of the Neolithic agricultural economy on the Tibetan Plateau ^54^. There might have been more than one expansion, or a series of movements, from Yellow River basin westwards, rather than a singular major Neolithic migration of millet farmers from Northwestern China into Tibetan Plateau and the Himalayas^55,56^. The low resolution of branching orders among Tibeto-Burman clades is expected given our wide sampling of languages distantly-related to the Sinitic clade, and reflects inherent uncertainties associated with reconstructing the evolutionary history of Sino-Tibetan languages.

Sino-Tibetan languages are distributed over most land areas of East Asia in a wide range of ecologies (e.g. lowland plain, mountains, basins, deserts, and high plateau)^57,58^. The sparse distribution of ethnolinguistic groups over Eastern China and the Tibetan plateau is in sharp contrast with the Himalayan region, one of the most linguistically diverse regions in the world and home to around 600 languages^59^. While major expansions of Sinitic and Bodish speakers had assimilated many earlier linguistic groups within in China^10,60^, the Himalayan region maintained high levels of ethnolinguistic diversity^61^, possibly as the result of stochastic drifts and long-term geographical isolation. The mountainous terrains of the Himalayan regions largely limited opportunities for social contact and cultural diffusion for groups living in close proximity^59^. Furthermore, the persisting semi-feudal political-economic system on the Tibetan Plateau may have facilitated social isolation among populations of different social status^62^ which could act as barriers for ethnolinguistic homogenisation. These geographic and social barriers are conducive to rapid cultural diversification^63^, which supports our finding that Himalayan subgroups are likely to have evolved independently despite their geographical proximity.

With a balanced sample representative of ethnolinguistic diversity (Table S6-8), our Sino-Tibetan phylogeny can be used for anthropological comparative analyses. Linguistic phylogenies can approximate the evolutionary history of cultural groups and are useful for studying the evolution of cultural traits against the background of cultural group descent. Since cultural groups descended from the same ancestral group are related historically, we cannot assume cultural differences as results of independent innovations without considering the descent of cultures. Phylogenetic comparative methods control for associations among cultural groups arising from their shared ancestry. They can be applied to estimate the ancestral states of a cultural trait, and test whether the transmission of cultural traits was functionally linked to particular ecological circumstances or geographical proximity^64^. Recent phylogenetic comparative studies have provided key insights into cultural evolution in Austronesian, Bantu, Indo-European, Pama-Nyungan and Uto-Aztecan populations^65^. Our reconstructed phylogeny has been applied to a quantitative cross-cultural database to study the cultural evolution of kinship and subsistence among Sino-Tibetan cultures (Ji et al*.*, forthcoming). The Himalayan region is one of the last refugia for ethnolinguistic diversity which have remained largely unexplored in cultural evolutionary studies. Future studies using our reconstructed phylogeny can elucidate the evolutionary history of Sino-Tibetan cultures.

## Materials and methods

### Language data

We compiled cognate data for basic vocabulary terms in 131 Sino-Tibetan languages (see also Table S6-S8 for coverage of major clades). The data are available from the *Tower of Babel* project (https://starling.rinet.ru/babel.php?lan=en), and are adapted from reconstructions by Peiros and Starostin^66^. We removed Bai due to high level of horizontal borrowing and Southern Chinese as it largely duplicates another Sinitic language in our sample (Beijing). The starling dataset comprises the Swadesh 100 word-list^67^ plus 10 additional concepts (far, heavy, near, salt, short, snake, thin, wind, worm, year). For reconstruction without the 10 additional concepts, see Figure S5. Loan words or borrowings are identified in the original dataset, and these were removed before performing phylogenetic inference. In some cases, more than one word was used to represent a particular meaning in a given language. These were coded as an additional binary trait for that meaning. This yielded a dataset in which each concept or meaning was treated as a single character with its associated cognates represented as multistate data; these multistate data were converted to presence/absence data to give a binary matrix coding for the presence (state = 1) or absence (state = 0) of 1726 cognate sets.

*Map of geographic distribution.* The scatter plot of sampled Sino-Tibetan language distribution was generated in Python v3.7.4 using the Plotly package v4.12.0 (https://plotly.com/python/). Geographic coordinates of language were accessed from the World Language Mapping System dataset^68^ and Ethnologue^69^.

### Phylogenetic inference

We inferred posterior distributions of phylogenetic trees using a Bayesian Markov-chain Monte Carlo (MCMC) inference framework applied to the binary data, and as implemented in the program *BEAST2*^23^*.* Bayesian methods allow users to sample trees and model parameters in proportion to their posterior probabilities, given the data, a model of cognate evolution and a set of prior beliefs about the distributions of model parameters and of the tree itself.

#### Models of cognate evolution

We compared several models of cognate evolution (Table S2) for their ability to describe the data: the simplest continuous-time Markov model that characterises rates of gains (0 → 1) and losses (1 → 0) of cognate classes (*m1p*), the *m1p* model augmented by gamma-distributed rate heterogeneity (with four rate categories)^70^, and the *m1p* model augmented by a binary covarion model (*CV*)^24^ that allows binary sites to switch *on* or *off* throughout the tree. The *m1p* model allows cognates to appear and disappear from a single language more than once over the course of time, mimicking the effect of word-borrowing and can accommodate a moderate level of horizontal transmission in the data^71^. We used exponentially distributed priors (mean = 10) on the transition rates.

#### Tree prior

We used the fossilised birth–death tree prior^22^ appropriate for time-structured data in which some taxa might not survive to the present. We modeled the proportion of sampled taxa (out of all languages in the family) with a uniform prior [0–1].

#### Inferring dated trees

We incorporated six time-calibrations on the tree (Table S1). Extinction timings of Old Chinese, Padam, and Shaiyang were calibrated with last-seen dates identified by linguists^66,72^; three internal nodes were calibrated based on historical records of the earliest observation of distinct descendant groups as the latest date of their most recent common ancestor. Dated trees were then inferred under a strict clock model and a model allowing for rates of evolution to vary among branches (so-called relaxed-clock model^73^). We used log-normally distributed rate variation in the relaxed-clock with μ = 1.0 and σ = 0.1.

#### Model selection

We used the stepping stone analysis implemented in BEAST2^23^ with 100 steps and 1 million samples per step to derive log marginal likelihoods of different evolution models. Table S2 shows log marginal likelihoods and log Bayes factors of all candidate models. The best fitting model is m1p augmented by binary covarion with relaxed clock. The relaxed-clock model with *m1p* + *CV* emerged as the best-supported model and we used this model to infer the final posterior sample of trees (Table S2).

#### MCMC chains

We ran at least five Markov chains with a burn-in period of 5,000,000 iterations and then allowed the chain to sample the posterior space for 50,000,000 iterations, sampling chains at intervals of 50,000 iterations to produce a posterior distribution of n = 900 trees with low average autocorrelation chains converged to the same regions of the parameter space and our final sample of 900 trees was drawn from one of these multiple chains. The maximum clade credibility tree was derived using TreeAnnotator v2.6.0^74^.

## Supplementary information

Supplementary Information.

## Data Availability

Nexus file of the posterior sample inferred using the best-fitting model is available in supplementary materials.
