# Publisher Correction: Dated phylogeny suggests early Neolithic origin of Sino-Tibetan languages

**DOI:** 10.1038/s41598-021-85112-w

**Published:** 2021-03-09

**Authors:** Hanzhi Zhang, Ting Ji, Mark Pagel, Ruth Mace

**Affiliations:** 1 Department of Anthropology, University College London, London, WC1H 0BW UK; 2 Key Laboratory of Animal Ecology and Conservation Biology, Centre for Computational and Evolutionary Biology, Institute of Zoology, Chinese Academy of Sciences, Beijing, 100101 China; 3 School of Biological Sciences, University of Reading, Reading, RG6 6UR UK; 4 Santa Fe Institute, Santa Fe, NM 87501 USA

Correction to: *Scientific Reports* https://doi.org/10.1038/s41598-020-77404-4, published online 27 November 2020

This Article contains an error where the nexus file of the posterior tree sample is missing from the Supplementary Information. This file appears below.

## Supplementary Information

Supplementary Information

