# Supplementary material for: Dated phylogeny suggests early Neolithic origin of Sino-Tibetan languages
Source: Sci Rep. 2020 Nov 27;10:20792. doi: 10.1038/s41598-020-77404-4 (PMC7695722; doi:10.1038/s41598-020-77404-4)
Supplement: Supplementary file 1 — Supplementary Information. [file 41598_2020_77404_MOESM1_ESM.docx]

Supplementary Information for

**Dated phylogeny suggests early Neolithic origin of Sino-Tibetan languages**

Hanzhi Zhang^1*^, Ting Ji^2^, Mark Pagel^3,4^ & Ruth Mace^1*^

^1^ Department of Anthropology, University College London, London WC1H 0BW, United Kingdom.

^2^ Key Laboratory of Animal Ecology and Conservation Biology, Centre for Computational and Evolutionary Biology, Institute of Zoology, Chinese Academy of Sciences, Beijing 100101, China

^3^ School of Biological Sciences, University of Reading, Reading RG6 6UR

^4^ Santa Fe Institute, Santa Fe, New Mexico 87501

^*^ Email: hanzhi.zhang.13@ucl.ac.uk; [r.mace@ucl.ac.uk](mailto:r.mace@ucl.ac.uk)

**This file includes:**

Supplementary text

Figures S1 to S5

Tables S1 to S8

SI References

**Other supplementary materials for this manuscript include the following:**

Nexus file of posterior tree sample

**Content**

# S1. Supplementary Method

## **S1.1 Cognate data.** We compiled cognate data for basic vocabulary terms in 131 Sino-Tibetan languages. The data are available from the *Tower of Babel* project (http:/starling.rinet.ru/ babel.php?lan=en), and are adapted from reconstructions by Peiros & Starostin^1^. We removed Bai due to high level of horizontal borrowing and Southern Chinese as it largely duplicates another Sinitic language in our sample (Beijing). The cognate data comprise the Swadesh 100 word-list^2^ plus 10 additional concepts (far, heavy, near, salt, short, snake, thin, wind, worm, year). Loan words or borrowings are identified in the original dataset, and these were removed before performing phylogenetic inference. This yielded a dataset in which each concept or meaning was treated as a single character with its associated cognates represented as multistate data; these multistate data were converted to presence/absence data to give a binary matrix coding for the presence (state=1) or absence (state=0) of 1726 cognate sets. In some cases, more than one word was used to represent a particular meaning in a given language. These were coded as an additional binary trait for that meaning.

## **S1.2 Phylogenetic time-calibration.** We incorporated six time-calibrations on the tree (Table S1). Three extinct languages were calibrated based on linguistic history; three internal nodes were calibrated based on historical records of the earliest observation of distinct descendant groups as the latest date of their most recent common ancestor. Dated trees were then inferred under a strict clock model and a model allowing for rates of evolution to vary among branches (so-called relaxed-clock model^3^). We used log-normally distributed rate variation in the relaxed-clock with μ=1.0 and σ=0.1. The relaxed-clock model with *m1p+CV* emerged as the best-supported model and we used this model to infer the final posterior sample of trees (Table S2).

**Table. S1 Time calibrations of ancestral nodes.**

| **Historical events** | **Time** | **Calibrations** | **References** |
| --- | --- | --- | --- |
| Extinction of  Old Chinese | 300 ~ 800 B.C. | Uniform [-2300, -2800] | Norman (1988)^4^ |
| Extinction of Padam | 1900 A.D. | -120 | Peiros & Starostin (1996)^1^ |
| Extinction of Shaiyang | 1900 A.D. | -120 | Peiros & Starostin (1996)^1^ |
| Lolo-Qiangic  common ancestor | Before 418 A.D. | Uniform [-1602, -∞] | “麽些蛮祖泥月乌逐出吐蕃”  *History of Yuan,* 1370 A.D. |
| Pumi  common ancestor | Before 1863 A.D. | Uniform [-157, -∞] | 基诺族普米族社会历史调查  Social History Survey of Jinuo Pumi (1990).^5^ |
| Naxi  common ancestor | Before 1723 A.D. | Uniform [-297, -∞] | 纳西族社会历史调查  Social History Survey of Naxi (1987).^6^ |

##

## **S1.3 Phylogenetic inference.** We inferred posterior distributions of phylogenetic trees using a Bayesian Markov-chain Monte Carlo (MCMC) inference framework applied to the binary data, and as implemented in the program *BEAST2*^7^*.* Bayesian methods allow users to sample trees and model parameters in proportion to their posterior probabilities, given the data, a model of cognate evolution and a set of prior beliefs about the distributions of model parameters and of the tree itself.

**S1.3.1 Models of cognate evolution.** We employed several models of cognate evolution (Figure S6), including the simplest continuous-time Markov model that characterise the rates of gains (0→1) and losses (1→0) of cognate classes (*m1p*), and the *m1p* model augmented by gamma-distributed rate heterogeneity (with four rate categories) ^8^, or/and a binary covarion model (*CV*)^9^ that allows binary sites to switch *on* or *off* throughout the tree. The *m1p* model allows a single cognate to appear and disappear from a single language more than once over the course of time, mimicking the effect of word-borrowing and can accommodate a moderate level of horizontal transmission in the data^10^. We used the stepping stone analysis implemented in BEAST2^7^ with 100 steps and 1 million samples per step to derive log marginal likelihoods of different evolution models. The best fitting model is m1p augmented by binary covarion with relaxed clock. We used exponentially distributed priors (mean = 10) on the transition rates. Dated trees were then inferred under a strict clock model and a model allowing for rates of evolution to vary among branches (so-called relaxed-clock model^3^). We used log-normally distributed rate variation in the relaxed-clock with μ=1.0 and σ=0.1. Table S2 shows that the relaxed-clock model with *m1p+CV* emerged as the best-supported model. Therefore, we employed this model to infer the final posterior sample of trees in all subsequent analyses.

**Table S2. Log marginal likelihoods of alternative cognate evolution models.** Model of cognate gain and loss (*m1p*) augmented by gamma rate heterogeneity with four rate categories (*G4*) or binary covarion (*CV*). Models of tree rate variation are strict clock (*strict*), relaxed clock with rates sampled from lognormal distribution (*relaxed*). Log marginal likelihoods were derived from stepping stone analyses implemented in BEAST2 with 64 steps and 1 million samples per step.

| **Cognate Evolution Model** | **Clock Model** | **Log Marginal Likelihood** | **Bayes Factor** |
| --- | --- | --- | --- |
| *m1p + CV* | relaxed | -28492.79 | - |
| *m1p + CV* | strict | -28525.19 | 65 |
| *m1p + G4* | relaxed | -31893.97 | 6802 |
| *m1p + G4* | strict | -31894.21 | 6803 |
| *m1p* | strict | -33849.32 | 10713 |
| *m1p* | relaxed | -33849.59 | 10714 |

###

**S1.3.2 Tree prior.** We used the fossilised birth-death tree prior^11^ appropriate for language evolution inferences. This allows us to include languages that have gone extinct before the present. The model also allows an estimate of the proportion of sampled taxa (out of all languages in the family) with a uniform prior [0-1].

**S1.3.3 Markov-chain Monte Carlo (MCMC) chains.** We ran at least five Markov chains with a burn-in period of 5,000,000 iterations and then allowed the chain to sample the posterior space for 50,000,000 iterations, sampling chains at intervals of 50,000 iterations to produce a posterior distribution of n=900 trees with low average autocorrelation chains converged to the same regions of the parameter space and our final sample of 900 trees was drawn from one of these multiple chains. The maximum clade credibility tree was derived using TreeAnnotator v2.6.0^12^.

##

**S1.4 Clade classification of reconstructed phylogeny**

- **Burmish:** Achang, Xiandao, Bola, Leqi, Langsu_Maru, Zhaiwa, Burmese
- **Yi:** Jinuo, Lijiang_Naxi, Ninglang_Naxi, Nusu, Rourou
- **Qiangic:** Muya, Queyu, Zhaba, Guiqiong, Namuzi, Shixing, North Qiang, South Qiang, South Pumi (Pumi1), North Pumi (Pumi2), Ersu, Lusu
- **Bodish:** Alike Tibetan, Xiaxe Tibetan, Lhasa Tibetan, Batang Tibetan, Written Tibetan, Jirel, Sherpa, Cuona Monpa, Motuo Monpa, Gurung, Tamang, Tamang, Thakali, Kaike
- **Naga:** Ang_Kohima, Ang_Khonoma, Chokri, Khezhama, Mao, Sema, Rengma, Kabui, Khoirao, Liangmai, Mzieme, Zeme, Maram, Tangkul, Ao_Chungli, Ao_Mongsen, Yimchungre, Sangtam, Lhota, Pochuri
- **Kuki-karbi:** Gangte, Tiddim, Haka, Hmar, Lushai, Mikir
- **Karenic:** Kelun, Sgaw, Palaichi, Geker, Kayan_Pekon, Yeinbaw, Padaung, Manu, W_Kayah, Yintale, Pa_O
- **Kho-Bwa:** Bugun, Sulong, Serdukpen
- **Bodo-Garo:** Bodo, Dimasa, Garo, Rabha
- **Northern Naga:** Chang, Konyak, Konyak-D, Phom, Nocte, Yogli, Wancho
- **Tani:** Apatani, Bokaw, Gallong, Hill_Miri, Tagin, Nishi, Upper_Bangni, Padam, Shaiyang, Milang
- **Kiranti:** Dumi, Khaling, Kulung, Sunwar, Limbu, Yamphe
- **Sinitic:** Beijing, Chinese (CHN), Fuzhou, Hakka, Old Chinese
- **Isolates:** Daofu_Horpa, Ergong, Gazhuo, Jiarung, Tujia, Anong, Dulong, Rawang, Miju_Geman, Manipuri, Chepang, Magar, Dhimal, Geba, Kham, Aka, Miji, Jingpo, Idu, Taraon, Kanauri, Lepcha

# S2. Supplementary Discussion

## **S2.1 Subgroup classification.** We found posterior support (>95% probability) for ten independent subgroups: Lolo-Burmese, Qiangic, Tibetic, Naga, Kuki-Karbi, Karenic, Kho-Bwa, Sal, Tani, Kiranti. Contrary to reported findings of the two previous studies, our inferences found no posterior support for Tibeto-Dulong, Tani-Idu, Tibeto-Gralrongic as independent subgroups.

We replicated the previous finding that Lolo-Burmese and Qiangic languages are closely related to each other (posterior probability = 0.95). We also replicate the finding that Bodo, Konyak, and Jingpo languages are closely related to each other and can be classified into a single subgroup ‘Sal’^13^. Nevertheless, our result showed very little support for the classification of Sal languages as a separate branch from all other Tibeto-Burman languages^14^.

Our reconstruction showed Kiranti languages form an independent subgroup (posterior probability = 0.92), and is unlikely to originate from the same ancestor as Magar, Kham, and Chepang (posterior probability = 0.02, see Table S2). Although previous phylogenetic study^15^ supported the classification of Tani language in the same subgroup with Idu, we failed to replicate this finding. We also found little support that Bodish and Lolo-Qiangic languages are closely related and form an independent subclade (posterior probability = 0.40), contrary to previous studies^15^.

**Table S3. Comparison of posterior probabilities of subgroups.** ‘ST-131’ refers to our reconstruction of 131 Sino-Tibetan languages without outgroup constraint, as shown in Figure S4)

| **Subgroups** | **ST-131** | **Zhang et al. 2019** | **Sagart et al. 2019** |
| --- | --- | --- | --- |
| **Tibeto-Burman** | 0.80 | 0.68 | 0.67 |
| **Lolo-Qiang** | 0.94 | 1 | 0.98 |
| **Lolo-Qiang-Tibetan** | 0.40 | 0.35 | 0.98 |
| **Tani-Idu** | 0.17 | 0.28 | 0.99 |
| **Kuki-Naga** | 0.69 | 0.92 | - |
| **Sal** | 1 | 0.99 | 0.96 |
| **“Himalayaish”**  (Chepang + Magar + Kiranti) | 0.02 | 0.78 | 0.12 |
| **Tibeto-Dulong** | 0.33 | 0.16 | 0.62 |

## **S2.2 Divergence time.** Previous phylogenetic studies inferred different time-depths for the initial divergence of the entire Sino-Tibetan phylogeny and the most recent common ancestor (MRCA) of key subgroups. Our inferences agreed with one of the previous phylogenetic study^15^ that Sinitic and Tibeto-Burman languages first began to diverge during the early Neolithic. However, the average time-depth of our reconstructed Sino-Tibetan phylogeny is around 8000 years BP, earlier than previous estimates. This is expected given our wider sample which includes Naga, Kho-Bwa, Karenic, Konyak languages that are distantly-related to Sinitic languages and were not included in previous phylogenetic studies.

|  | **ST-131** | **Zhang et al. 2019** | **Sagart et al. 2019** |
| --- | --- | --- | --- |
| **Initial divergence** | 8.0 [4.8 ~ 11.8] | 5.9 [4.2 ~ 7.8] | 7.2 [5.1 ~ 9.6] |
| Age of Subgroup MRCA | | | |
| **Subgroups** | **ST-131** | **Zhang et al. 2019** | **Sagart et al. 2019** |
| Lolo-Qiangic | 3.2 [1.9~4.7] | 2.8 [2.2~3.4] | 4.3 [3.0~5.7] |
| Tibetan-Qiangic | 4.1 [2.5~6.1] | 3.9 [3.3~5. 1] | 4.8 [3.3~6.3] |
| Kiranti | 2.1 [1.2~3.2] | 2.8 [1.8~3.9] | 3.6 [2.4~5.1] |
| Tani-Idu | 5.0 [2.5~7.1] | 4.0 [3.5~5.3] | 4.1 [2.4~5.8] |
| Sal | 3.9 [2.1~5.8] | 3.4 [2.5~4.6] | 4.7 [3.1~6.7] |
| Kuki-Karbi | 4.5 [2.5~6.6] | - | 5.0 [3.4~6.7] |
| Karenic | 1.3 [0.7~1.9] | 1.1 [1.0~1.3] | - |
| Naga | 2.6 [1.6~4.0] | 2.7 [1.9~3.5] | - |
| Sinitic | 2.6 | 2.7 [2.5~3.0] | 2.6 |

**Table S4. Comparison of clade- and root-age estimates and 95% Highest posterior density (HPD) with two previous studies, in thousand-years.** ‘ST-131’ refers to our reconstruction of 131 Sino-Tibetan languages with fixed Sinitic outgroup, as shown in Figure 2.

**
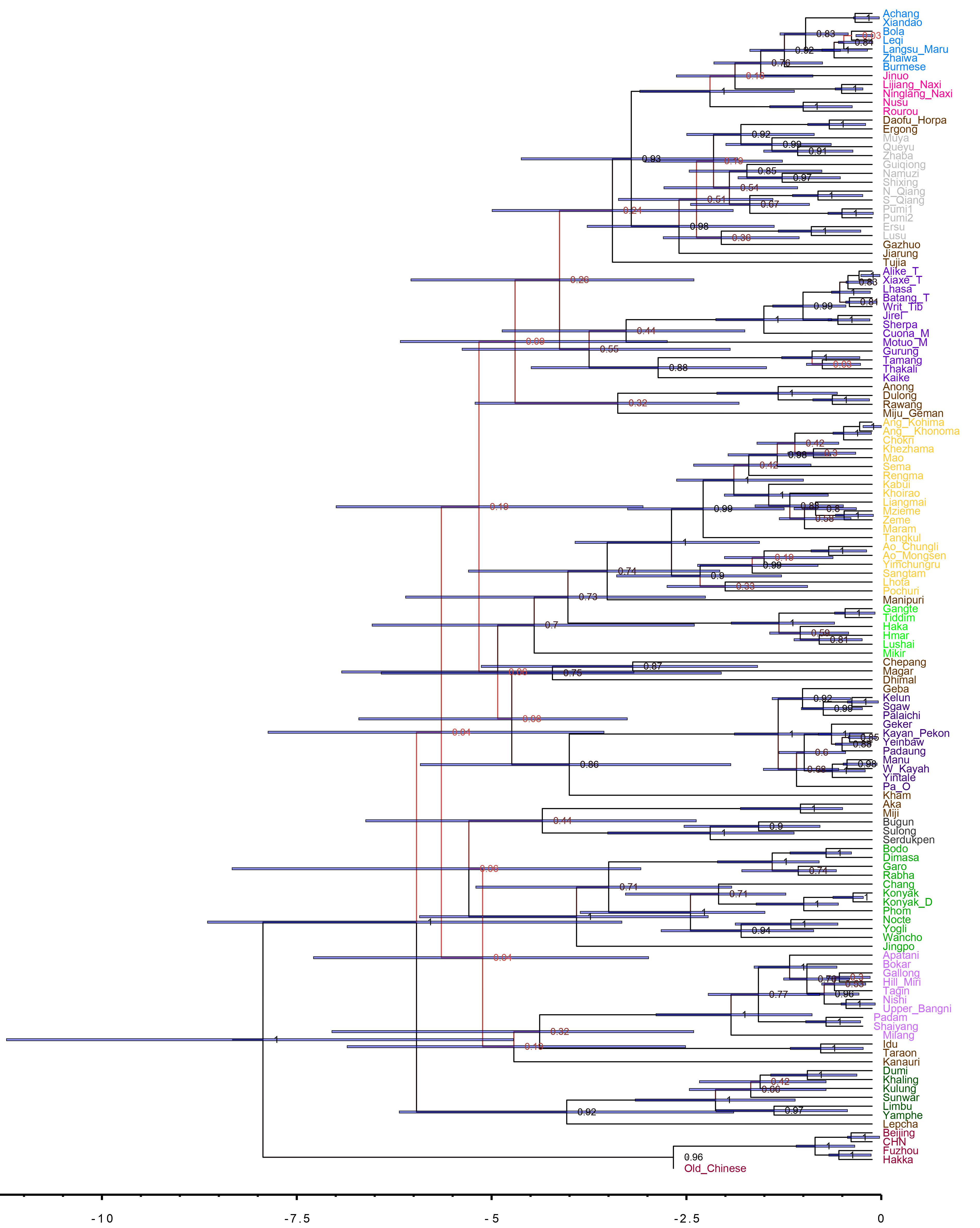
**

**Fig. S1. Maximum Clade Credibility phylogeny showing 95% HPD node heights, reconstructed using relaxed-clock + covarion model and fixed Sinitic Outgroup.** Posterior probability of internal nodes is shown in numner and branch colours (Red: lowest posterior probability; Black: highest posterior probability). Time scale in units of thousand-year.

**
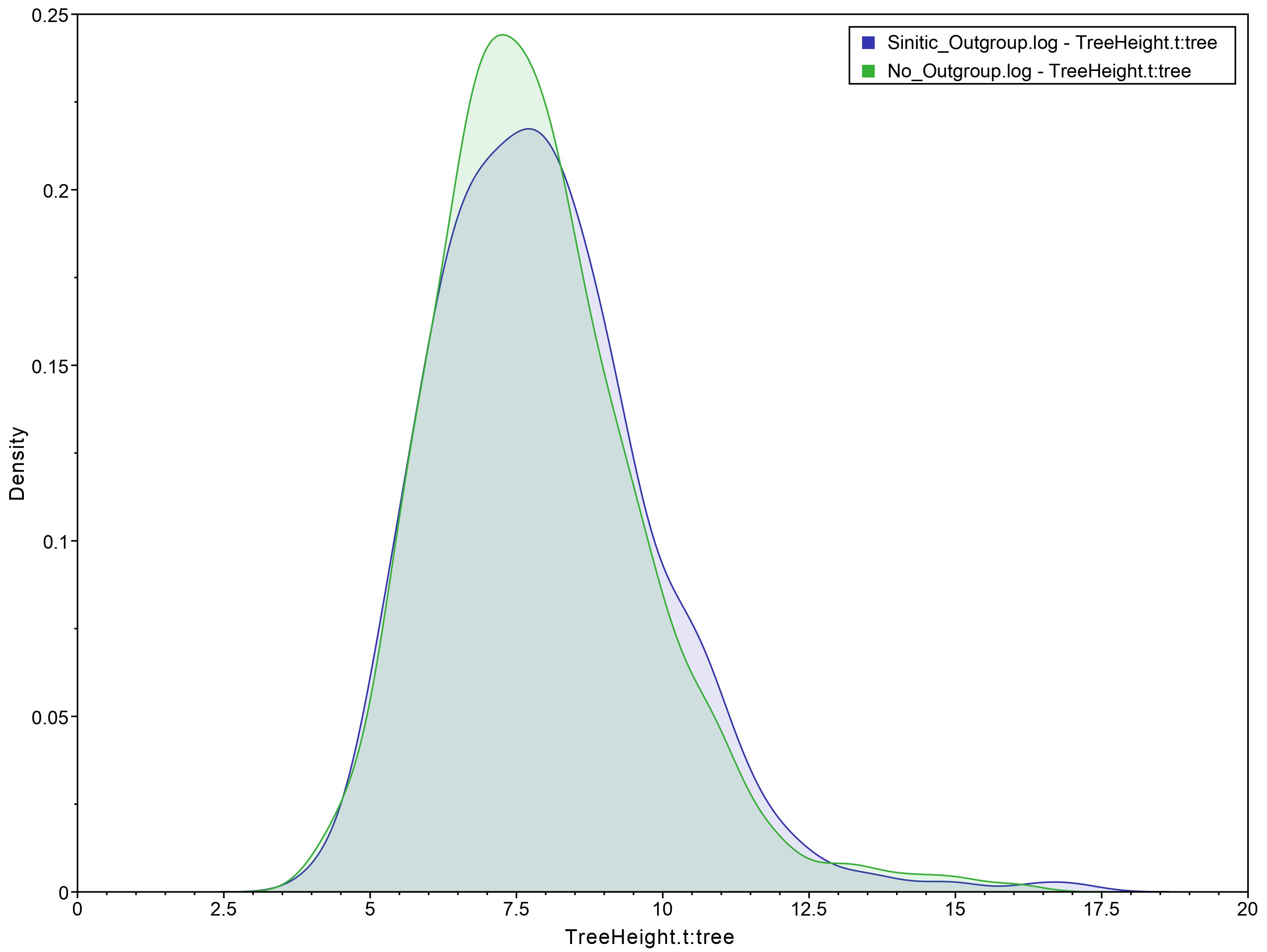
**

**Fig. S2. Kernel density estimation of Sino-Tibetan phylogeny time depth (in thousand-year) inferred using relaxed-clock and covarion model, with and without outgroup constraint.** Mean estimates of inferences without outgroup constraint is 7878 (95% HPD = 4079 ~ 11112). Mean estimates of inferences with fixed Sinitic outgroup is 7983 (95% HPD = 4778 ~ 11285).


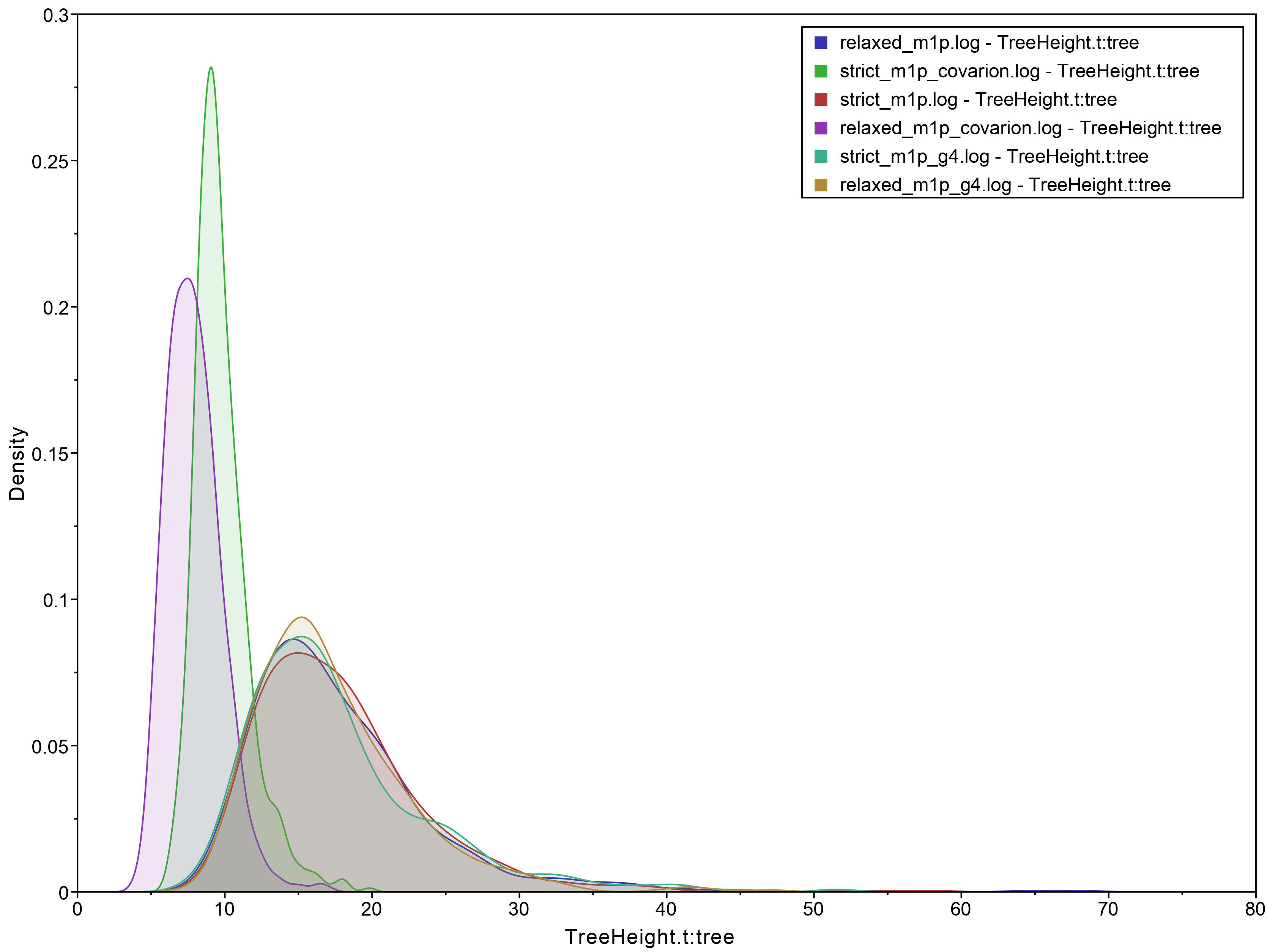
**Figure S3. Root age inferred with alternative cognate evolution models.** Time scale in units of thousand-year. Model of cognate gain and loss (*m1p*) augmented by gamma rate heterogeneity with four rate categories (*g4*) or binary covarion (*covarion*). Models of tree rate variation are strict clock (*strict*), relaxed clock with rates sampled from lognormal distribution (*relaxed*). The mean root age estimated with a relaxed clock and covarion model is 7983 BP (95% HPD = 4778 ~ 11285). The mean root age estimated with a strict clock and covarion model is 9758 BP (95% HPD= 6648 ~13465).

## **S2.3 Outgroup.** To identify the outgroup of Sino-Tibetan phylogeny, we first performed inferences on our data without any monophyletic constraint. Similar to the two previous studies ^15,16^, we found the Sinitic clade forms the most plausible outgroup with 80.13% posterior probability, followed by the second candidate (Sinitic+Sal+Tani-Idu+Kho-Bwa clades) with a much lower posterior probability of 15.65%. Table S5 show that the marginal likelihood of inferences with Sinitic outgroup constraint are higher than inferences without any outgroup constraint. There is strong evidence that the model with the Sinitic outgroup constraint is better-fitting than the model without any outgroup constraint. We present the Maximum Clade Credibility tree of inferences without any outgroup constraint in Figure S5**.**

**Table S5.** Log marginal likelihood of phylogenetic reconstructions without any outgroup constraint and with specifying Sinitic clade as the outgroup. Both inferred with the most likely evolution model (relaxed-clock + m1p + binary covarion). Log marginal likelihoods were derived from stepping stone analyses implemented in BEAST2 with 100 steps and 1 million samples per step.

| **Outgroup constraint** | **Log marginal likelihood** | **Bayes Factor** |
| --- | --- | --- |
| No outgroup constraint | -28468.32 | 20.18 |
| Sinitic outgroup | -28458.23 | - |

**
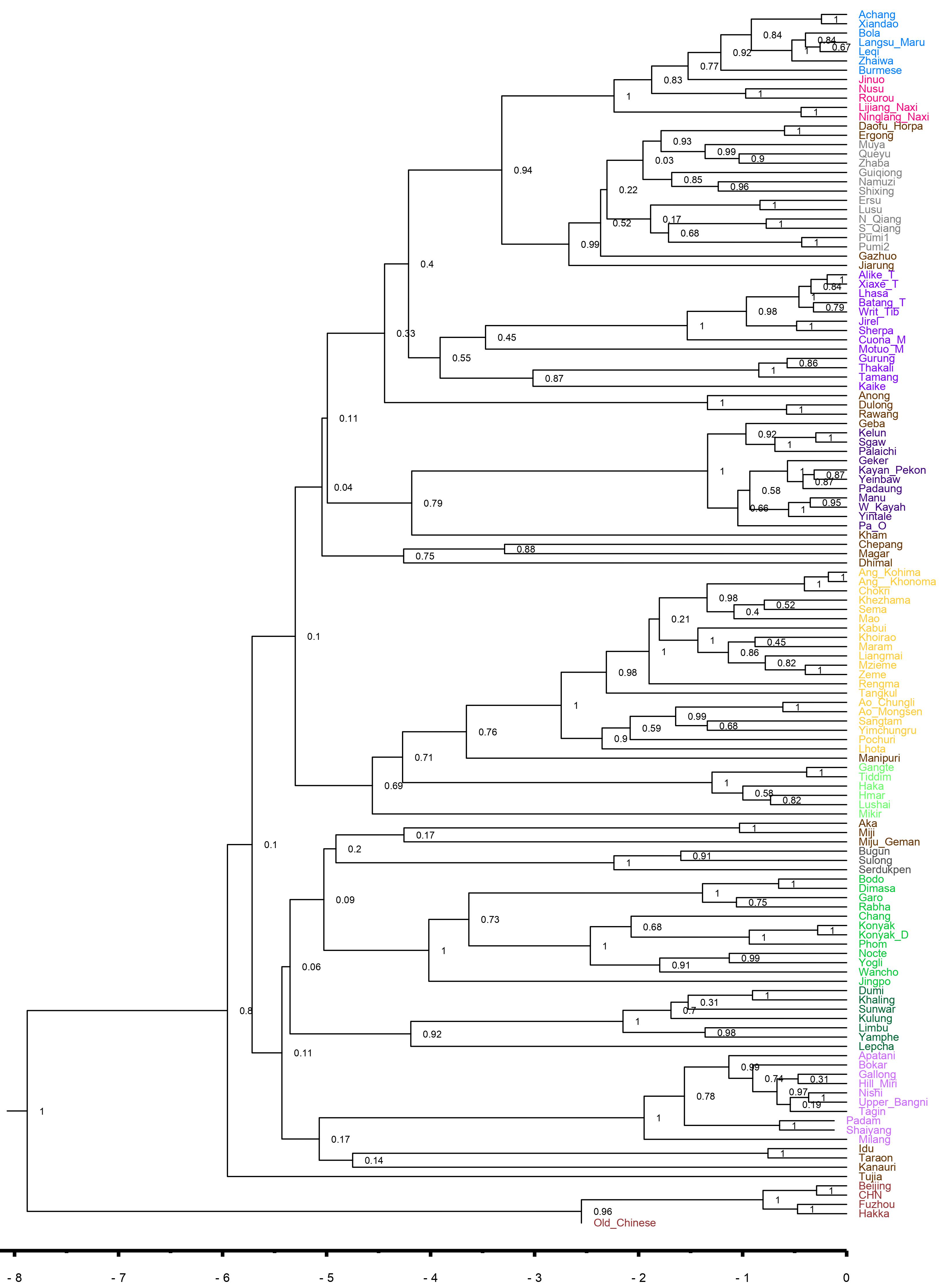
**

**Figure S4. Maximum Clade Credibility phylogeny reconstructed using relaxed-clock + covarion model without outgroup constraint.** Posterior probabilities of internal nodes are shown. Time scale in units of thousands-of-years.

## **S2.4 Sample coverage.**

**Table S6. Sample size of major clades.** Number of sampled languages in major Sino-Tibetan clades in our study (‘ST-131’) versus previous studies.

| **Major Clades** | **ST-131** | **Zhang et al. 2019** | **Sagart et al. 2019** |
| --- | --- | --- | --- |
| Lolo-Qiangic | 26 | 48 | 15 |
| Tibetan-Qiangic | 39 | 60 | 20 |
| Kiranti | 6 | 5 | 7 |
| Tani-Idu | 5 | 5 | 3 |
| Sal | 12 | 9 | 3 |
| Kukichin-Karbi | 6 | 6 | 4 |
| Karenic | 12 | 3 | 0 |
| Naga | 20 | 10 | 0 |
| Sinitic | 5 | 2 | 7 |

**Table S7. Tree balance**. Percentage of sampled languages in each clade**.** Our sample has a lower variance (i.e. standard deviation) of sampling proportions across clades and is more balanced than previous studies.

| **Major Clades** | **ST-131** | **Zhang et al. 2019** | **Sagart et al. 2019** |
| --- | --- | --- | --- |
| Lolo-Qiangic | 20 | 44 | 30 |
| Tibetan-Qiangic | 30 | 55 | 40 |
| Kiranti | 5 | 5 | 14 |
| Tani-Idu | 4 | 5 | 6 |
| Sal | 9 | 8 | 6 |
| Kukichin-Karbi | 5 | 6 | 8 |
| Karenic | 9 | 3 | 0 |
| Naga | 15 | 9 | 0 |
| Sinitic | 4 | 2 | 14 |
| **Standard Deviation** | **8.91** | **19.75** | **13.61** |

| **Major Clades** | **ST-131** | **Zhang et al. 2019** | **Sagart et al. 2019** |
| --- | --- | --- | --- |
| Lolo-Qiangic | 19 | 36 | 11 |
| Tibetan-Qiangic | 18 | 27 | 9 |
| Kiranti | 18 | 15 | 21 |
| Tani-Idu | 56 | 56 | 33 |
| Sal | 32 | 24 | 8 |
| Kukichin-Karbi | 11 | 11 | 7 |
| Karenic | 57 | 14 | 0 |
| Naga | 74 | 37 | 0 |
| Sinitic | 31 | 13 | 44 |
| **Mean** | **35.11** | **25.89** | **14.78** |

**Table S8. Sampling coverage.** Percentage of all recorded languages on Ethnologue ^17^ for each clade covered in our sample and in previous studies. Sampling of previous studies were not incorporated because multistate data of coded cognate sets are not readily available. On average, our sample covers a higher proportion of all known languages in each clade than previous studies.

**
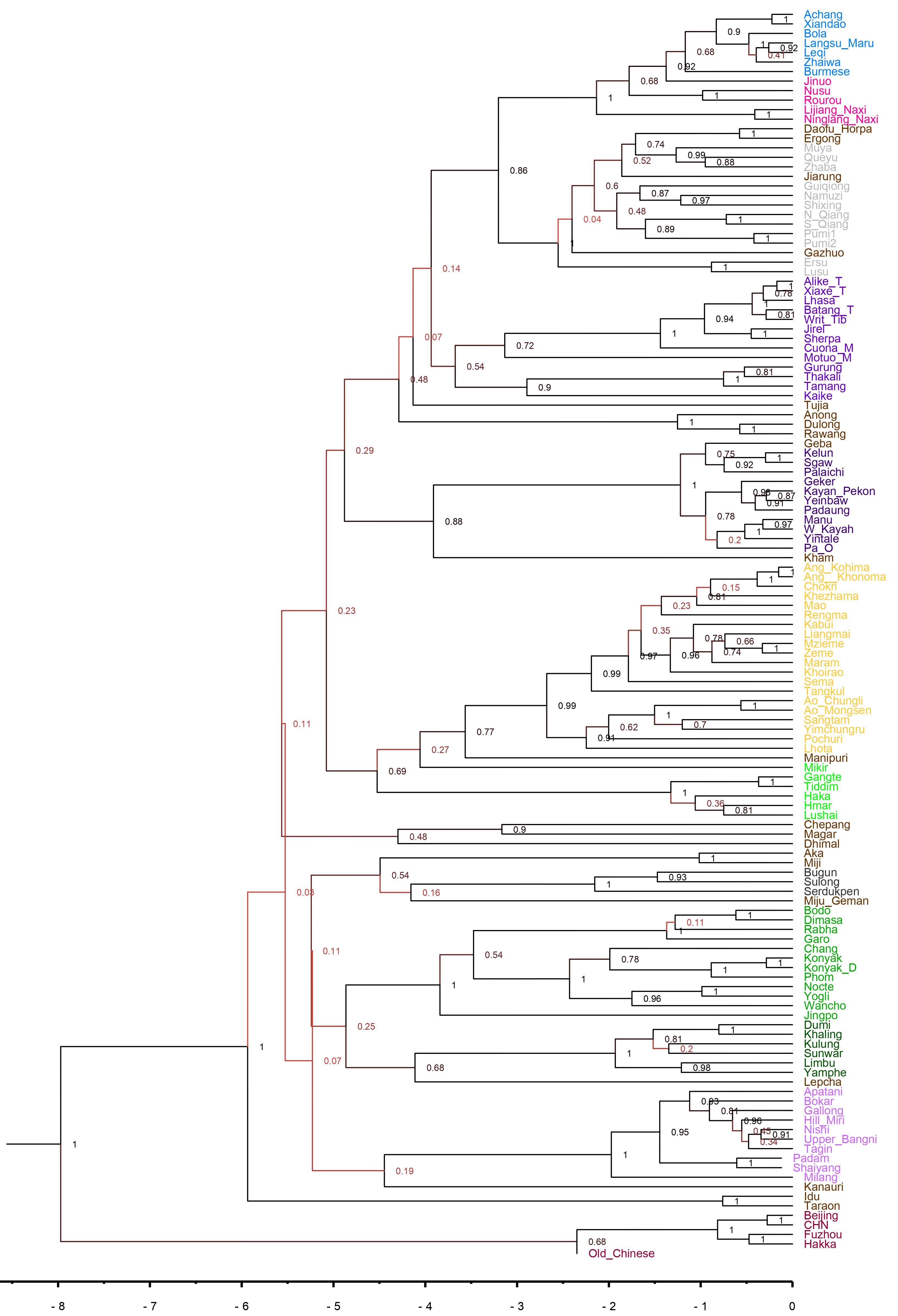
Figure S5. Maximum Clade Credibility phylogeny reconstructed using cognate-sets of Swadesh-100 concepts only (without 10 additional concept), reconstructed using relaxed-clock + covarion model and fixed Sinitic Outgroup.** Posterior probabilities of internal nodes are shown. Time scale in units of thousands-of-years. Mean root age = 7972 years BP (95% HPD = [4475 ~ 12362]).

# Supplementary References

1 Peiros, I. & Starostin, S. *A comparative vocabulary of five Sino-Tibetan languages*. (The University of Melbourne, Department of Linguistics and Applied Linguistics, 1996).

2 Swadesh, M. The origin and diversification of languages. *Aldine Transactions, New Brunswick/Londres* (1971).

3 Drummond, A. J., Ho, S. Y., Phillips, M. J. & Rambaut, A. Relaxed phylogenetics and dating with confidence. *PLoS biology* **4**, e88 (2006).

4 Norman, J. *Chinese*. (Cambridge University Press, 1988).

5 云南省编辑委员会. *基诺族普米族社会历史综合调查*. (民族出版社, 1990).

6 四川省编辑组. *四川省纳西族社会历史调查*. (四川省社会科学院出版社, 1987).

7 Bouckaert, R. *et al.* BEAST 2: a software platform for Bayesian evolutionary analysis. *PLoS computational biology* **10**, e1003537 (2014).

8 Yang, Z. Maximum likelihood phylogenetic estimation from DNA sequences with variable rates over sites: approximate methods. *Journal of Molecular evolution* **39**, 306-314 (1994).

9 Penny, D., McComish, B. J., Charleston, M. A. & Hendy, M. D. Mathematical elegance with biochemical realism: the covarion model of molecular evolution. *J Mol Evol* **53**, 711-723, doi:10.1007/s002390010258 (2001).

10 Atkinson, Q., Nicholls, G., Welch, D. & Gray, R. From words to dates: water into wine, mathemagic or phylogenetic inference? *Transactions of the Philological Society* **103**, 193-219 (2005).

11 Heath, T. A., Huelsenbeck, J. P. & Stadler, T. The fossilized birth–death process for coherent calibration of divergence-time estimates. *Proceedings of the National Academy of Sciences* **111**, E2957-E2966 (2014).

12 Rambaut, A. & Drummond, A. TreeAnnotator v. 2.3. 0. Part of the BEAST package. (2014).

13 Burling, R. The sal languages. *Linguistics of the Tibeto-Burman Area* **7**, 1-32 (1983).

14 Benedict, P. K. Sino-Tibetan: another look. *Journal of the American Oriental Society*, 167-197 (1976).

15 Sagart, L. *et al.* Dated language phylogenies shed light on the ancestry of Sino-Tibetan. *Proc Natl Acad Sci U S A* **116**, 10317-10322, doi:10.1073/pnas.1817972116 (2019).

16 Zhang, M., Yan, S., Pan, W. & Jin, L. Phylogenetic evidence for Sino-Tibetan origin in northern China in the Late Neolithic. *Nature* **569**, 112-115, doi:10.1038/s41586-019-1153-z (2019).

17 Simons, G. F. *Ethnologue: Languages of the world*. (sil International, 2017).
